# Supplementary material for: An integrative view of the regulatory and transcriptional landscapes in mouse hematopoiesis
Source: Genome Res. 2020 Mar;30(3):472–84. doi: 10.1101/gr.255760.119 (PMC7111515; doi:10.1101/gr.255760.119)
Supplement: Supplemental Material [file supp_30_3_472__index.html]

An integrative view of the regulatory and transcriptional landscapes in mouse hematopoiesis — Supplemental Material 

# An integrative view of the regulatory and transcriptional landscapes in mouse hematopoiesis

## Supplemental Material

- Supplemental\_Results\_Figures\_Methods\_R9.docx
- Supplemental\_Table\_S1.xlsx
- Supplemental\_Table\_S2.xlsx
- Supplemental\_Table\_S3.xlsx
- Supplemental\_Table\_S4.xlsx
- Supplemental\_Table\_S5.xlsx
- Supplemental\_Code.zip
