# Supplementary figures and images for: An integrative view of the regulatory and transcriptional landscapes in mouse hematopoiesis
Source: Genome Res. 2020 Mar;30(3):472–84. doi: 10.1101/gr.255760.119 (PMC7111515; doi:10.1101/gr.255760.119)

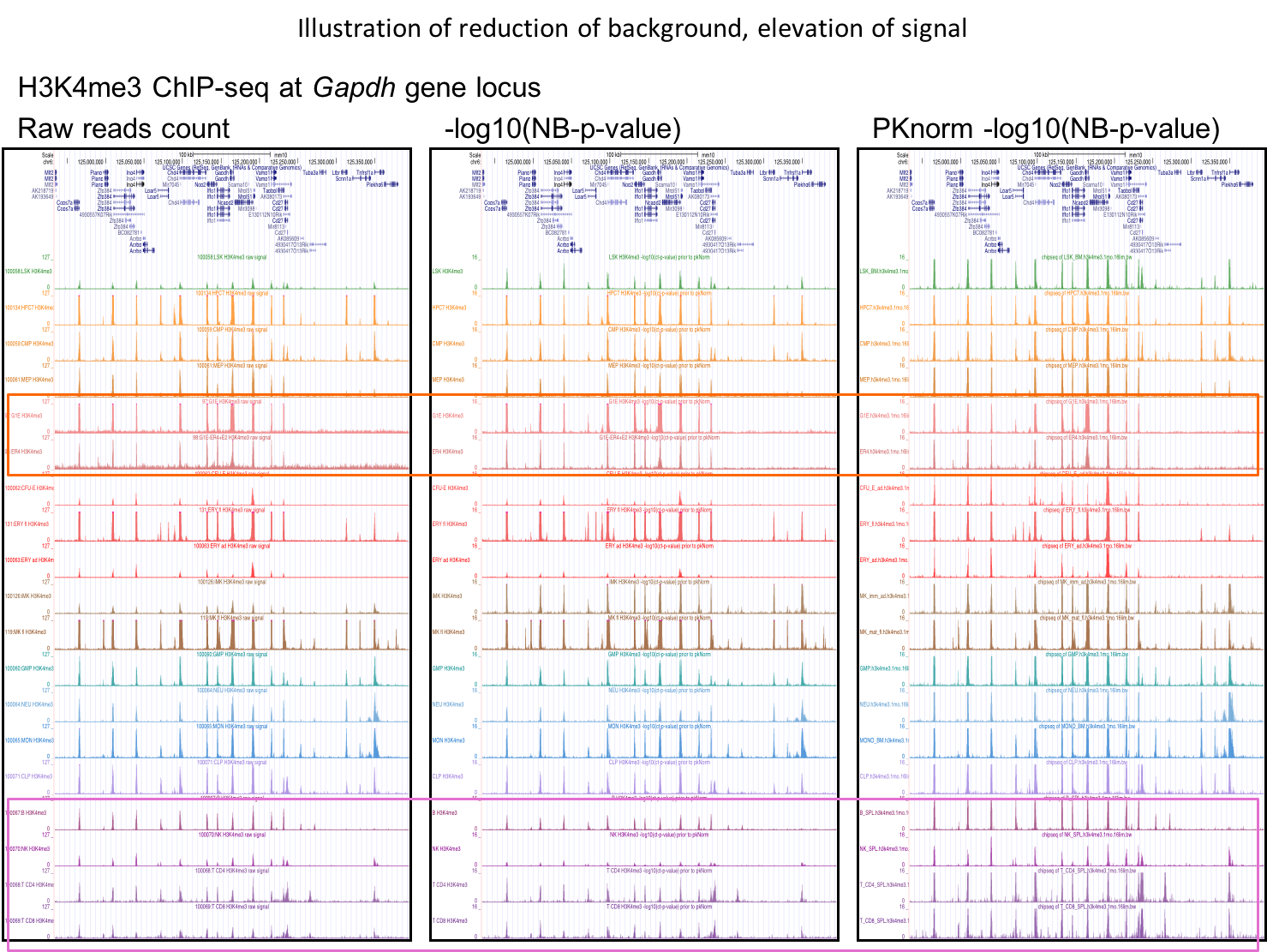

Supplement: Supplemental Material [file supp_gr.255760.119_Supplemental_Code.zip › Supplemental_Code/01_S3norm_pipeline/example_figures/rc2pknorm.png]

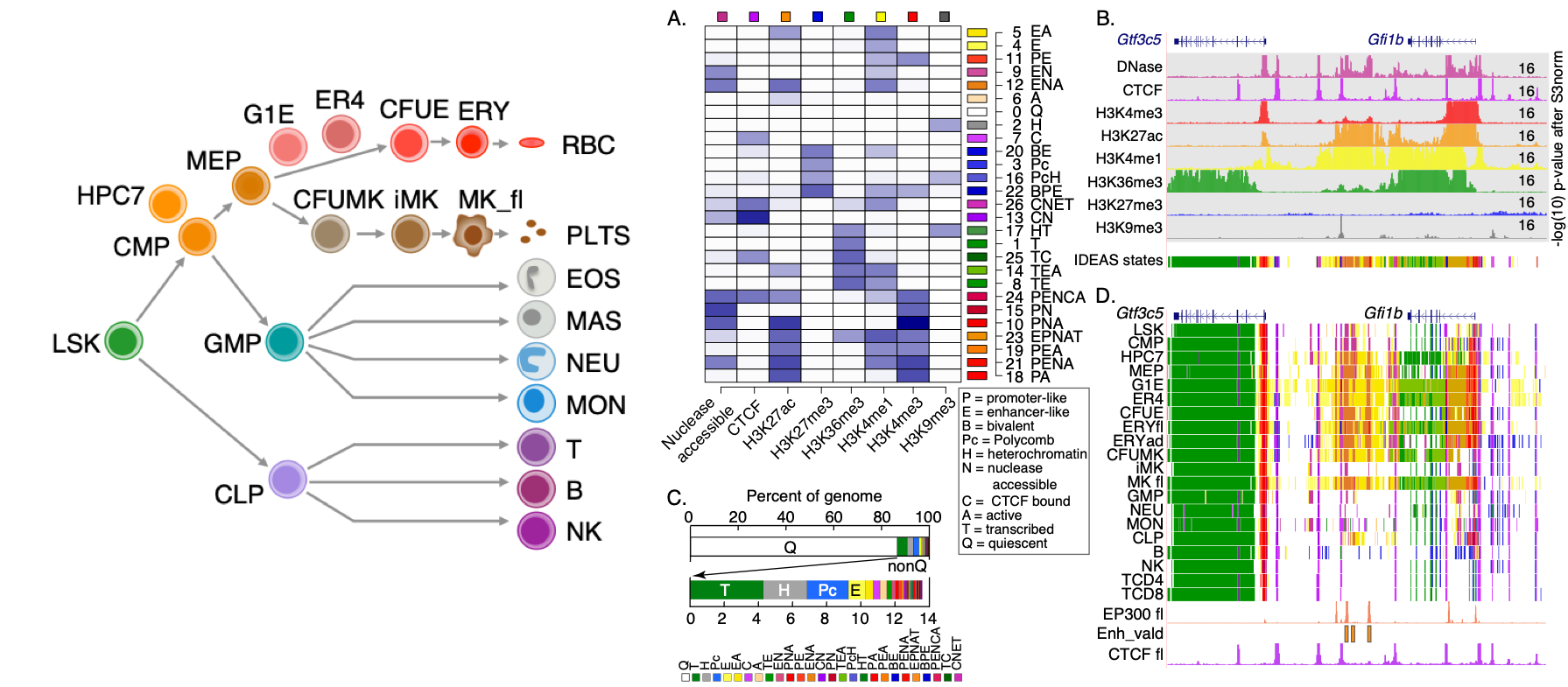

Supplement: Supplemental Material [file supp_gr.255760.119_Supplemental_Code.zip › Supplemental_Code/figures_for_github/vision_mouse.png]
